# Editorial: The evolution of biomineralization in metazoans

**DOI:** 10.3389/fgene.2022.1092695

**Published:** 2023-01-04

**Authors:** Mélanie Debiais-Thibaud, Frédéric Marin, Sylvain Marcellini

**Affiliations:** ^1^ Institut des Sciences de l’Evolution de Montpellier, ISEM, Univ Montpellier, CNRS, IRD, EPHE, Montpellier, France; ^2^ Laboratoire Biogéosciences, UMR CNRS-EPHE 6282, Université de Bourgogne—Franche-Comté, Dijon, France; ^3^ Laboratory of Development and Evolution (LADE), University of Concepción, Concepción, Chile

**Keywords:** biomineralisation, evolution, metazoan, corals, mollusks, vertebrates, sponges (porifera)

Biomineralization refers to the process by which living systems deposit minerals. This process has shaped the face of the Earth over geological time, since the Archean with the appearance of the first stromatolites around 3.5 billion years ago, but particularly with the almost simultaneous emergence of multiple biologically-controlled animal mineralizations - both skeletal and non-skeletal—at the dawn of the Cambrian times, circa 545 million year ago (Knoll, 2003). Metazoan calcium carbonate, calcium phosphate and silica became then major actors of the Earth machine: let us think for a moment that Australia’s Great Barrier Reef is the only animal construction visible from space ! Throughout the Phanerozoic times, these three minerals have contributed to maintain Earth homeostasis and, concerning calcium carbonate, to regulate the climate at global scale, by long-term sequestering carbon dioxide (Milliman, 1993).

This Frontiers Research Topic gathers fifteen articles that bring new insights into biomineralization in many metazoan phyla, highlighting both the great diversity of mechanisms as well as some ancient evolutionary events that shaped this important biological process. Figure 1 illustrates the variety of contributions covering a large range of metazoan phyla. Of course, encompassing the whole metazoans was a challenge and some groups are missing in this volume: siliceous sponges for example, but also brachiopods, bryozoans, calcifying annelids, or, among the ecdysozoans, crustaceans. However, the fifteen articles presented here constitute a significant sampling of the research on evolutionary processes that have been driving animal biomineralization for an eon.

**FIGURE 1:**
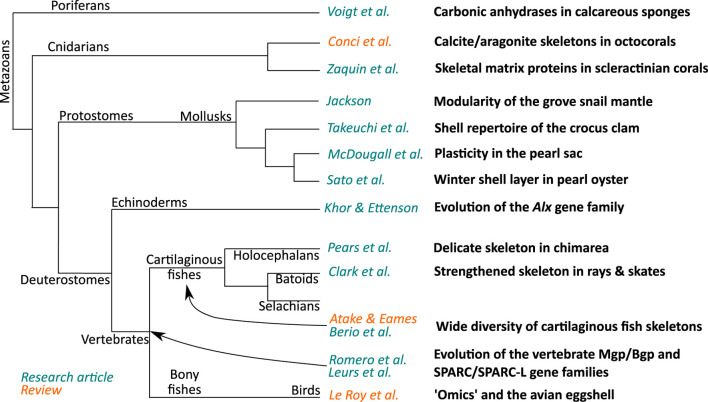
The phylogenetic relationships of the 15 contributions of this Research Topic.

Regarding non-bilaterians metazoans, Voigt et al. highlight the evolution of carbonic anhydrases in calcareous sponges (Calcarea), distinguishing the different cellular locations of this enzyme in the two subclasses Calcinea and Calcaronea. For cnidarians, Zaquin et al. show how the skeletal matrix proteins of scleractinian corals result from the independent co-option of ancestral genes predating cnidarian diversification, but also from posterior recruitment of duplicated genes that are species-specific. Finally, Conci et al. review different cellular and molecular aspects of the skeletal biomineralization of another cnidarian clade, Octocorallia, which can ‘handle’ the two calcium carbonate polymorphs, *i.e.*, calcite—the predominant form—but also, more rarely, aragonite.

The four papers covering the protostome world deal with the molecular aspects of shell biomineralization in molluscs, namely, three bivalves and one gastropod. Sato et al. emphasize the seasonal plasticity of the shell, by describing the microstructure of the winter layer and its associated molecular markers, in the shell of the Japanese pearl oyster, while McDougall et al. associate changes in gene expression in the pearl sac to the pearl structural characteristics in the south sea pearl oyster *Pinctada maxima*. Fundamental aspects of shell evolution are examined with the contribution of Takeuchi et al. who identify the complete protein shell repertoire of the Japanese crocus clam. At last, Jackson reveals the modularity and plasticity of the calcifying mantle of the terrestrial grove snail, *Cepaea nemoralis*, by looking at the spatial expression pattern of some key shell-forming genes.

A total of eight articles relate to biomineralisation in the skeleton of deuterostomes, with a major focus on the derived calcium-phosphate based skeleton of vertebrates. In particular, Pears et al. describe in unprecedented detail the delicate mineralisation in the cartilaginous skeleton of Holocephalans, while Clark et al. uncover the various architectures of highly mineralised jaws in batoids (rays). More general aspects of cartilaginous fish skeletal mineralisation are exposed in the review of Atake and Eames and a research article proposed by Berio et al. Specific aspects of skeletal gene evolution are analysed for two vertebrate gene families: *Mgp/Bgp* Leurs et al., and *SPARC/SPARCL* Romero et al., Deuterostomian calcium carbonate-based biomineralizations are not to be outdone, with the contribution of Le Roy et al. on the molecular toolkit for building avian eggshell and with the review of Khor and Ettenson on the skeletal function of Alx homeobox genes in echinoderms and all deuterostomes in general.

While the Research Topic of these research and review articles demonstrates the diversity of biomineralization forms in animals, they also surprisingly underline major genetic variation at all levels of comparisons, from the deepest nodes of metazoans, to more recent divergent groups within vertebrates, molluscs, corals or sponges. We hope that this Research Topic will inspire and encourage the community to further explore this complex and fascinating aspect of animal biology.

## References

[B1] Knoll A. H. (2003). Biomineralization and evolutionary history. Biominer. Rev. Mineral. Geochem. 54, 329–356. 10.2113/0540329

[B2] Milliman J. D. (1993). Production and accumulation of calcium carbonate in the ocean: Budget of a nonsteady state. Glob. Biogeochem. Cycles 7, 927–957. 10.1029/93gb02524

